# Outcomes Following Discectomy for Far Lateral Disc Herniation Are Not Predicted by Obstructive Sleep Apnea

**DOI:** 10.7759/cureus.14921

**Published:** 2021-05-09

**Authors:** John Connolly, Austin J Borja, Svetlana Kvint, Donald K. E Detchou, Gregory Glauser, Krista Strouz, Scott D McClintock, Paul J Marcotte, Neil R Malhotra

**Affiliations:** 1 Department of Neurosurgery, University of Pennsylvania Perelman School of Medicine, Philadelphia, USA; 2 McKenna EpiLog Fellowship in Population Health, University of Pennsylvania, Philadelphia, USA; 3 Department of Mathematics, West Chester University, West Chester, USA

**Keywords:** outcomes, hospital readmissions, far lateral disc herniation, discectomy, stop-bang

## Abstract

Introduction

Previous studies have demonstrated that obstructive sleep apnea (OSA) is associated with adverse postoperative outcomes, but few studies have examined OSA in a purely spine surgery population. This study investigates the association of the STOP-Bang questionnaire, a screening tool for undiagnosed OSA, with adverse events following discectomy for far lateral disc herniation (FLDH).

Methods

All adult patients (n = 144) who underwent FLDH surgery at a single, multihospital, academic medical center (2013-2020) were retrospectively enrolled. Univariate logistic regression was performed to evaluate the relationship between risk of OSA (low- or high-risk) according to STOP-Bang score and postsurgical outcomes, including unplanned hospital readmissions, ED visits, and reoperations.

Results

Ninety-two patients underwent open FLDH surgery, while 52 underwent endoscopic procedures. High risk of OSA according to STOP-Bang score did not predict risk of readmission, ED visit, outpatient office visit, or reoperation of any kind within either 30 days or 30-90 days of surgery. High risk of OSA also did not predict risk of reoperation of any kind or repeat neurosurgical intervention within 30 days or 90 days of the index admission (either during the same admission or after discharge).

Conclusion

The STOP-Bang questionnaire is not a reliable tool for predicting post-operative morbidity and mortality for FLDH patients undergoing discectomy. Additional studies are needed to assess the impact of OSA on morbidity and mortality in other spine surgery populations.

## Introduction

Complications following surgery are a significant driver of patient dissatisfaction and healthcare costs [1, 2]. This is especially pertinent to spine surgeries, due to the increasing case volume and the risk for costly, and potentially avoidable, adverse outcomes [3, 4]. In an effort to incentivize health systems and providers to prevent postoperative complications, the Centers for Medicare & Medicaid Services (CMS) and commercial payers have implemented incentive-based payment systems, where reimbursements are at least partially based on quality metrics such as readmission [5, 6]. These payment systems include CMS’s Bundled Payments for Care Improvement (BPCI) program, which reimburses health systems a fixed, risk-adjusted payment for care associated with a specific medical episode or procedure [7]. Such quality-based payment modalities lead to equivalent or decreased healthcare costs, while maintaining or improving quality metrics, relative to fee-for-service payment modalities [8, 9]. In spine surgery, hospitals participating in the BPCI Advanced program had lower readmission rates following cervical spine surgery, lumbar fusion, and lumbar discectomy [10].

As public and private payers alike transition to quality-based payment systems for spine surgery, it is crucial for providers to identify patients at an elevated risk for potentially experiencing postoperative complications. Numerous factors have been associated with complications following spine surgery, including patient age, operative duration, and medical comorbidities [11-13]. However, there remains a need for validated tools to predict adverse postoperative events.

One potential screening tool is the STOP-Bang questionnaire, which is amongst the most accepted methods to estimate a patient’s risk of undiagnosed obstructive sleep apnea (OSA) [14-17]. This survey may be easily and routinely administered in the preoperative clinic setting and quickly accessed from any electronic health record (EHR) system. OSA is associated with adverse postoperative events, particularly pulmonary and cardiac complications, across many surgical disciplines [14-16]. Notably, over 80% of sleep apnea cases are undiagnosed, indicating that the STOP-Bang questionnaire may be an important tool for risk stratifying patients not previously known to have OSA [18]. The STOP-Bang questionnaire has been validated to project post-surgical complications among patients undergoing craniotomy for supratentorial brain tumors [19, 20]. However, there exists no research that assesses the predictive value of this tool on postoperative outcomes in purely spine surgery populations.

In the present study, we assess how the binary risk of OSA, assessed by the STOP-Bang questionnaire score, affects postoperative outcomes following surgery for far lateral disc herniation (FLDH). Compared to other forms of disc herniation, FLDH responds less readily to conservative management. Additionally, far lateral lumbar discectomy is technically difficult due to the difficulty in accessing the interpedicular compartment without damaging the nerve root or inducing dural tear [21, 22]. Moreover, FLDH is amenable to both open and minimally invasive, endoscopic surgical approaches [23]. Therefore, risk stratification among this population of spine surgery patients may be particularly useful for perioperative planning.

## Materials and methods

Sample selection

The present study retrospectively enrolled consecutive adult patients (n = 144) who underwent far lateral lumbar discectomy FLDH repair at a single, multi-hospital, 1659-bed, university health system from 2013 to 2020. Data were acquired from open FLDH surgeries (n = 92) from July 1, 2013 to April 30, 2020, and from endoscopic operations (n = 52) from June 1, 2017 to April 30, 2020 (endoscopic procedures were not performed at this institution before June 2017). All patients completed the STOP-Bang questionnaire preoperatively. The Institutional Review Board considered this study to be of minimal risk to patients and granted a waiver of informed consent. Patient information and procedural variables were captured using the EpiLog tool, a non-proprietary data acquisition software created by the senior author of the present study and layering into the institution’s existing EHR system [24].

Data acquisition

Using the acquired patient data, STOP-Bang score was determined for each patient (Table 1). Additional information - including gender, race, insurance type, operating time (from initial surgical incision to the completion of multilayer wound closure), and body mass index (BMI) - was utilized to control for confounding variables during univariate logistic regression. Adverse postoperative events were recorded, including unplanned readmissions, emergency department (ED) visits, neurosurgery outpatient office visits, and reoperations of any kind during the 30-day (30D) and 30-90-day (30-90D) post-surgical windows. Additionally, reoperations of any kind and repeat neurosurgical operations were determined within 30D and 90-days (90D) of the index admission (either during the index admission or post-discharge).

**Table 1:** STOP-Bang Score Questionnaire Components of the STOP-Bang questionnaire. Higher STOP-Bang score predicts greater risk of undiagnosed obstructive sleep apnea (OSA). Scores range from 0 to 8, with scores 2 and below representing low-risk, and scores 3 and above representing high-risk.

		Question
Patient Characteristic	Snore	Do you snore loudly?
	Tiredness	Do you often feel tired during the daytime?
	Observed apnea	Has anyone seen you stop breathing during your sleep?
	Pressure	Do you have or are you being treated for high blood pressure?
	BMI	Is your BMI > 35 kg/m^2^
	Age	Are you older than 50 years?
	Neck	Is your neck size large? (>43 cm for men, 41 cm for women)
	Gender	Are you male?

Statistical analysis

Categorical variables are presented as frequencies (percent), and continuous variables are reported as means with standard deviations. Univariate regression was used to assess the predictive value of STOP-Bang score, evaluated on a binary scale (STOP-Bang score ≤2 corresponded to low-risk of OSA; ≥3 corresponded to high-risk of OSA), for adverse outcomes. A significant result was defined as p < 0.05. All endpoint analysis was executed via SAS Version 9.4 (SAS Institute Inc., Cary, NC).

## Results

Patient characteristics

In all patients who underwent FLDH surgery (n = 144), mean age was 61.72 ± 11.55 years, and mean STOP-Bang score was 2.54 ± 1.50 (Table 2). Sixty-nine (47.9%) patients were female, and 126 patients (87.5%) were non-Hispanic white. Mean American Society of Anesthesiologists (ASA) Grade was 2.30 ± 0.52, and mean BMI was 28.49 ± 4.83 kg/m^2^. In the open FLDH surgery subgroup (n = 92), mean age was 60.24 ± 11.36 years, and mean STOP-Bang score was 2.16 ± 1.35. Forty-one (44.6%) patients were female, and 81 patients (88.0%) were non-Hispanic white. Mean ASA Grade was 2.25 ± 0.48, and mean BMI was 28.37 ± 4.89 kg/m^2^. In the endoscopic FLDH repair subgroup (n = 52), mean age was 64.35 ± 11.42 years, and mean STOP-Bang score was 3.21 ± 1.75. Twenty-eight (53.9%) patients were female, and 45 patients (86.5%) were non-Hispanic white. Mean ASA Grade was 2.38 ± 0.57, and mean BMI was 28.71 ± 4.70 kg/m^2^.

**Table 2:** Patient Characteristics Overview of characteristics for patients who underwent far lateral lumbar discectomy.

		All FLDH, n = 144	Open FLDH, n = 92	Endoscopic FLDH, n = 52
Patient Characteristic	STOP-Bang score, mean (sd)	2.54 (1.50)	2.16 (1.35)	3.21 (1.75)
	Age, mean (sd)	61.72 (11.55)	60.24 (11.36)	64.35 (11.42)
	Gender, n (%)			
	Male	75 (52.08)	51 (55.43)	24 (46.15)
	Female	69 (47.92)	41 (44.57)	28 (53.85)
	Race, n (%)			
	White	126 (87.50)	81 (88.04)	45 (86.52)
	Non-White	18 (12.50)	11 (11.96)	7 (13.46)
	Household income ($), mean (sd)	78,283 (26,996)	78,807 (27,784)	77,356 (25,515)
	Insurance Type, n (%)			
	Commercial	24 (16.67)	16 (17.39)	8 (15.38)
	Government	59 (40.97)	34 (36.96)	26 (0.00)
	Managed Care	59 (40.97)	41 (44.57)	18 (34.62)
	Worker’s Compensation	1 (0.69)	1 (1.09)	0 (0)
	BMI (kg/m^2^), mean (sd)	28.49 (4.83)	28.37 (4.89)	28.71 (4.70)
	ASA Grade, mean (sd)	2.30 (0.52)	2.25 (0.48)	2.38 (0.57)
	Operating time (min), mean (sd)	63.79 (41.04)	83.49 (39.94)	35.37 (13.48)

FLDH surgery outcomes

**Figure 1:**
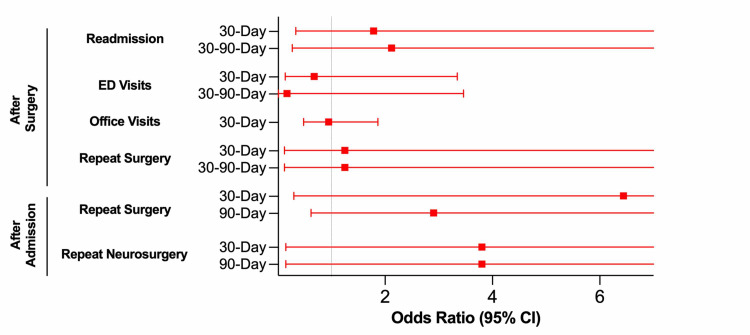
OSA and STOP-Bang - Overall FLDH Outcome Prediction Odds ratios and 95% confidence intervals for patients undergoing any FLDH surgery (significance set at p = 0.05). OR > 1.0 correlates with higher risk of outcome for patients with higher risk for OSA; OR < 1.0 correlates with lower risk of outcome for patients with higher risk for OSA. OSA: Obstructive sleep apnea; FLDH: Far lateral disc herniation.

High risk of OSA according to STOP-Bang score did not predict risk of readmission or ED visit following FLDH surgery in either the 30D or 30-90D post-surgery windows (Table 3, Figure 1). STOP-Bang score did not predict neurosurgery outpatient office visit rates 30D after surgery. STOP-Bang score did not predict risk of reoperation of any kind in either the 30D or 30-90D post-surgery windows. STOP-Bang score also did not predict risk of reoperation of any kind or risk of repeat neurosurgical intervention 30D or 90D following the index admission (either during the same admission or after discharge).

**Table 3:** Patient Outcomes and Complications Logistic regression to examine the impact of obstructive sleep apnea (OSA) risk as assessed by STOP-Bang score on postsurgical outcomes. Bolded values denote statistical significance (p < 0.05). OR = Odds ratio; CI = Confidence interval.

		All FLDH, n = 144	Open FLDH, n = 92	Endoscopic FLDH, n = 52
Post-Surgery Outcomes	30D Readmission	n = 5 (3.47%)	n = 4 (4.45%)	n = 1 (1.92%)
		OR 1.787	OR 2.123	OR 1.657
		95% CI 0.337 – 9.477	95% CI 0.34 – 13.248	95% CI 0.059 – 46.239
		p = 0.50	p = 0.42	p = 0.77
	30-90D Readmission	n = 3 (2.08%)	n = 2 (2.17%)	n = 1 (1.92%)
		OR 2.12	OR 2.085	OR 1.657
		95% CI 0.269 – 16.72	95% CI 0.202 – 21.508	95% CI 0.059 – 46.239
		p = 0.48	p = 0.54	p = 0.77
	30D ED Visits	n = 6 (4.17%)	n = 3 (3.26%)	n = 3 (5.77%)
		OR 0.68	OR 0.279	OR 1.062
		95% CI 0.138 – 3.345	95% CI 0.013 – 5.834	95% CI 0.09 – 12.58
		p = 0.64	p = 0.41	p = 0.96
	30-90D ED Visits	n = 3 (2.08%)	n = 2 (2.17%)	n = 1 (1.92%)
		OR 0.172	OR 0.397	OR 0.169
		95% CI 0.009 – 3.462	95% CI 0.018 – 8.928	95% CI 0.006 – 4.631
		p = 0.25	p = 0.56	p = 0.29
	30D Office Visits	n = 55 (38.19%)	42 (45.65%)	n = 13 (25.00%)
		OR 0.948	OR 1.296	OR 1.26
		95% CI 0.482 – 1.867	95% CI 0.541 – 3.107	95% CI 0.328 – 4.847
		p = 0.88	p = 0.56	p = 0.74
	30D Reoperation	n = 2 (1.39%)	n = 2 (2.17%)	n = 0 (0.00%)
		OR 1.252	OR 2.086	N/A
		95% CI 0.125 – 12.527	95% CI 0.202 – 21.508	N/A
		p = 0.85	p = 0.54	N/A
	30-90D Reoperation	n = 2 (1.39%)	n = 1 (1.09%)	n = 1 (1.92%)
		OR 1.252	OR 0.672	OR 1.657
		95% CI 0.125 – 12.527	95% CI 0.025 – 17.798	95% CI 0.059 – 46.239
		p = 0.85	p = 0.81	p = 0.77
Post-Admission Outcomes	30D Reoperation	n = 2 (1.39%)	n = 1 (1.09%)	n = 1 (1.92%)
		OR 6.44	OR 6.356	OR 1.657
		95% CI 0.298 – 139.316	95% CI 0.243 – 166.145	95% CI 0.059 – 46.239
		p = 0.24	p = 0.27	p = 0.77
	90D Reoperation	n = 7 (4.86%)	n = 3 (3.26%)	n = 4 (7.69%)
		OR 2.903	OR 1.231	OR >999
		95% CI 0.62 – 13.578	95% CI 0.151 – 10.041	95% CI <0.001 – >999
		p = 0.18	p = 0.85	p = 0.96
	30D Neurosurgical Reoperation	n = 1 (0.69%)	n = 1 (1.09)	n = 0 (0.00%)
		OR 3.803	OR 6.356	N/A
		95% CI 0.149 – 96.987	95% CI 0.243 – 166.145	N/A
		p = 0.42	p = 0.27	N/A
	90D Neurosurgical Reoperation	n = 1 (0.69%)	n = 1 (1.09%)	n = 0 (0.00%)
		OR 3.803	OR 6.356	N/A
		95% CI 0.149 – 96.987	95% CI 0.243 – 166.145	N/A
		p = 0.42	p = 0.27	N/A

Open FLDH surgery outcomes

**Figure 2:**
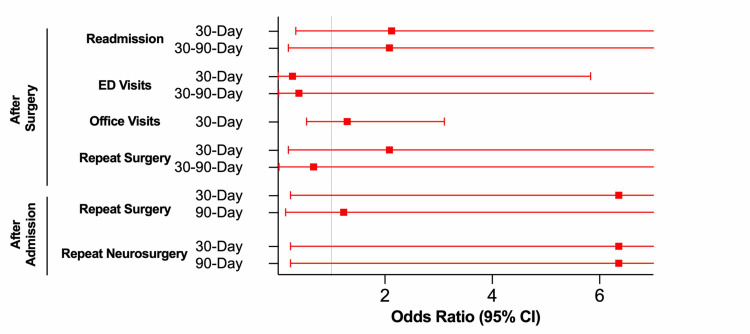
OSA and STOP-Bang - Open FLDH Outcome Prediction Odds ratios and 95% confidence intervals for patients undergoing open FLDH surgery (significance set at p = 0.05). OR > 1.0 correlates with higher risk of outcome for patients with higher risk for OSA; OR < 1.0 correlates with lower risk of outcome for patients with higher risk for OSA. FLDH: Far lateral disc herniation; OSA: Obstructive sleep apnea.

STOP-Bang score did not predict risk of readmission or ED visit following open FLDH surgery in either the 30D or 30-90D post-surgery windows (Table 3, Figure 2). STOP-Bang score did not predict neurosurgery outpatient office visit rates 30D after surgery. STOP-Bang score did not predict risk of reoperation of any kind in either the 30D or 30-90D post-surgery windows. STOP-Bang Score also did not predict risk of reoperation of any kind or risk of repeat neurosurgical intervention 30D or 90D following the index admission (either during the same admission or after discharge).

Endoscopic FLDH surgery outcomes

**Figure 3:**
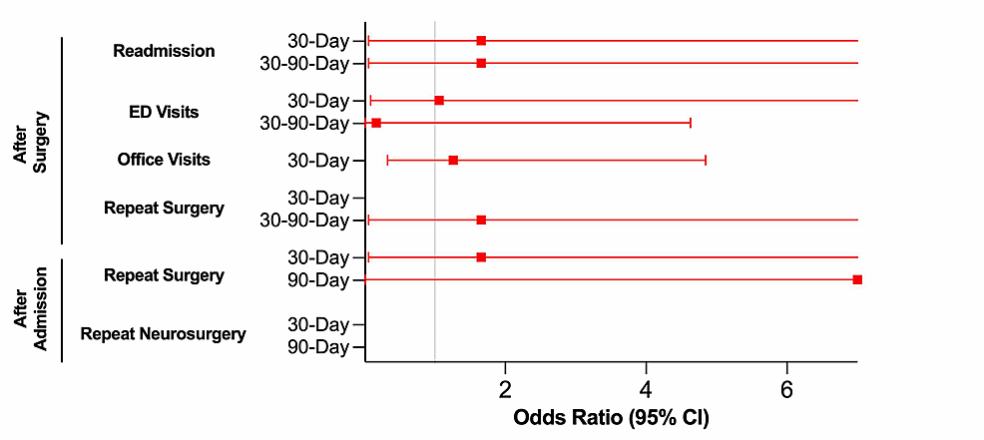
OSA and STOP-Bang - Endoscopic FLDH Outcome Prediction Odds ratios and 95% confidence intervals for patients undergoing endoscopic FLDH surgery (significance set at p = 0.05). OR > 1.0 correlates with higher risk of outcome for patients with higher risk for OSA; OR < 1.0 correlates with lower risk of outcome for patients with higher risk for OSA. OSA: Obstructive sleep apnea; FLDH: Far lateral disc herniation.

STOP-Bang score did not predict risk of readmission or ED visit following endoscopic FLDH surgery in either the 30D or 30-90D post-surgery windows (Table 3, Figure 3). High risk of OSA did not predict neurosurgery outpatient office visit rates 30D after surgery. STOP-Bang score did not predict risk of reoperation of any kind in either the 30D or 30-90D post-surgery windows. STOP-Bang score also did not predict risk of reoperation of any kind or risk of repeat neurosurgical intervention 30D or 90D following the index admission (either during the same admission or after discharge).

## Discussion

The present analysis employed univariate logistic regression to compare binary risk of OSA to rates of adverse postoperative outcomes. Assessed on a binary scale, risk of OSA as determined by the STOP-Bang questionnaire is not associated with postoperative outcomes, including readmissions, ED visits, and reoperations, following discectomy for FLDH. These findings suggest that use of the STOP-Bang questionnaire for risk stratification may not be justified for patients undergoing FLDH surgery. Although previous studies have validated the STOP-Bang questionnaire as a tool for predicting untoward postoperative outcomes in neurosurgical populations, here we found no evidence that suggests that higher STOP-Bang scores were correlated with unfavorable patient outcomes.

Healthcare payment reform has crystallized the importance of preventing postoperative complications. No longer do complications only affect patients - they also hold negative financial consequences for health systems, who may see reimbursement rates decline under quality-based payment modalities should they fail to prevent complications [7]. Risk stratification ought to synthesize demographic variables, procedural variables, and medical comorbidities in order to provide individualized postoperative care optimized to prevent unfavorable complications. In the case of spine surgeries, providers may wish to consider patient age, insurance status, surgical history, length of surgery, and comorbidities in planning postoperative care [25].

Risk-stratifying patients, and identifying patients at elevated risk of suffering complications, holds promise as a method for focusing care efforts on high-risk patients. Surgeons and affiliated care teams may wish to engage high-risk patients in programs aimed at preventing readmissions and reoperations [25-27]. These programs may include at-home nursing care following surgery, or at-home social work visits [28]. Moreover, risk stratifying patients may allow providers to allocate postoperative care resources more efficiently between low-risk and high-risk patients. In doing so, health systems may reduce health system costs (through abrogation of excess care to low-risk patients) while increasing quality performance (through decreased complication rates of high-risk patients), thereby benefiting both patient and health system financial wellbeing. This is particularly important for surgeries as serious and technically complex as far lateral discectomy, which is associated with higher risk of adverse events [21].

In spine surgery, anesthesia risk factors such as OSA inform surgical approach and peri- and intraoperative anesthesia technique [29, 30]. That is, patients with a higher risk of OSA may represent riskier candidates for open repairs and general anesthesia. However, while the STOP-Bang score has proven to be a useful tool for care quality improvement efforts in other neurosurgical populations, the results herein suggest it may not be applicable to FLDH repair, a relatively short procedure. In contrast, OSA may play a more significant role in more extensive operations with longer recovery periods, such as multi-level spinal fusion.

In this study, we preliminarily demonstrate that binary STOP-Bang score is not useful in predicting outcomes of FLDH repair. Based on this result, providers may eschew binary STOP-Bang score as a metric for risk stratification, and allocation of postoperative care resources, for patients undergoing procedures for FLDH. However, as the STOP-Bang questionnaire is validated for use in other neurosurgical populations, as well as other surgical populations, further research is required into the association between OSA and outcomes following spine procedures.

## Conclusions

OSA has been associated with adverse peri- and postoperative outcomes across multiple surgical specialties, including neurosurgery. Metrics that predict OSA, such as the STOP-Bang questionnaire, are enticing tools that allow surgeons to risk-stratify patients. In the present study, high risk for OSA as determined by the STOP-Bang score was not associated with adverse postoperative outcomes or increased health system utilization for FLDH patients undergoing discectomy. Further research into the association of undiagnosed OSA, as predicted by the STOP-Bang score, with morbidity and mortality in spine surgery populations is warranted.
